# Long memory mean and volatility models of platinum and palladium price return series under heavy tailed distributions

**DOI:** 10.1186/s40064-016-3768-y

**Published:** 2016-12-09

**Authors:** Edmore Ranganai, Sihle Basil Kubheka

**Affiliations:** Department of Statistics, University of South Africa, Cnr Christiaan de Wet and Pioneer Avenue, Florida Park, Roodepoort, 1710 South Africa

**Keywords:** Platinum, Palladium, Long memory, Structural breaks, Volatility, Heavy tailed distribution, Heteroskedasticity, ARFIMA–FIGARCH type models

## Abstract

South Africa is a cornucopia of the platinum group metals particularly platinum and palladium. These metals have many unique physical and chemical characteristics which render them indispensable to technology and industry, the markets and the medical field. In this paper we carry out a holistic investigation on long memory (LM), structural breaks and stylized facts in platinum and palladium return and volatility series. To investigate LM we employed a wide range of methods based on time domain, Fourier and wavelet based techniques while we attend to the dual LM phenomenon using ARFIMA–FIGARCH type models, namely FIGARCH, ARFIMA–FIEGARCH, ARFIMA–FIAPARCH and ARFIMA–HYGARCH models. Our results suggests that platinum and palladium returns are mean reverting while volatility exhibited strong LM. Using the Akaike information criterion (AIC) the ARFIMA–FIAPARCH model under the Student distribution was adjudged to be the best model in the case of platinum returns although the ARCH-effect was slightly significant while using the Schwarz information criterion (SIC) the ARFIMA–FIAPARCH under the Normal Distribution outperforms all the other models. Further, the ARFIMA–FIEGARCH under the Skewed Student distribution model and ARFIMA–HYGARCH under the Normal distribution models were able to capture the ARCH-effect. In the case of palladium based on both the AIC and SIC, the ARFIMA–FIAPARCH under the GED distribution model is selected although the ARCH-effect was slightly significant. Also, ARFIMA–FIEGARCH under the GED and ARFIMA–HYGARCH under the normal distribution models were able to capture the ARCH-effect. The best models with respect to prediction excluded the ARFIMA–FIGARCH model and were dominated by the ARFIMA–FIAPARCH model under Non-normal error distributions indicating the importance of asymmetry and heavy tailed error distributions.

## Background

South Africa is a country rich in the platinum group metals (PGMs) particularly platinum and palladium and it is the largest producer of platinum and second largest producer of palladium (Matthey 2014) accounting for 96% of known PGMs global reserves. In addition to accounting for a significant proportion of global mineral production and resources, the contribution of the PGMs to South Africa economically and otherwise cannot be over-emphasized. For instance, on average from 2008 to 2013, the percentage contribution to the South African GDP from this sector was 2.3% with a yearly increase of 3.3% and a head count of 191 781 in direct employment. Further, PGMs also play significant roles in the investment arena (Batten et al. 2010). Since platinum and palladium are two of the major precious metals that offer different volatility and returns of lower correlations with stocks at both sector and market levels, they are some of the attractive asset classes eligible for portfolio diversification (Arouri et al. 2012) which appear more likely to act as a financial instrument than gold. Recently, palladium has entered the Johannesburg Securities Exchange (JSE) as exchange traded funds (ETF). Two palladium funds, Standard Bank AfricaPalladium ETF and Absa Capital newPalladium ETF have been launched in March of 2014 on the JSE. These exchange traded funds are backed by the physical palladium metal. Also, the roles of the PMGs in the the medical field (e.g., their use in anticancer complexes) and industrial catalysis are ever-advancing. Given this background, investigating the mechanisms which generate these data returns and their related dynamics are of paramount importance to policy makers, regulators, traders and investors globally.

It is well known that financial returns and hence volatility are dominated by the stylized facts. These include nonstationarity, volatility clustering, their returns are not normally distributed, i.e., the empirical distributions are more peaked and heavy tailed and sometimes asymmetrical and the autocorrelation functions (ACFs) of squared (absolute) returns and volatility exhibit persistence. Further, in precious metals returns and volatility, evidence of their respective ACFs exhibiting a hyperbolic decay, a phenomenon referred to as long memory (LM) (long range dependence) rather than an exponential one (short memory) exists in the literature. The LM phenomenon may be coupled with structural breaks which are shown to severely compromise LM tests as structural breaks induce spurious LM (Baneree and Urga 2005). Recent events that could result in structural breaks in the PGMs returns and volatility are the 2008/2009 global financial crisis and the occasional mining industry labour unrest since the 2012 Marikana incident which resulted in the death of 34 miner during a nation-wide labour unrest. Such events bring extremes and jumps in data that may alter the underlying data generating mechanisms.

In the literature nonconstant variance (heteroskedasticity) is handled by autoregressive heteroskedastic (ARCH) models (Engle 1982) and generalized ARCH (GARCH) models (Bollerslev 1986) while LM in the mean is handled by autoregressive fractionally integrated moving average (ARFIMA) models (Tsay 2002). LM can be also inherent in the volatility and fractionally integrated GARCH (FIGARCH) models (Baillie et al. 1996) are proposed as appropriate models. ARFIMA and FIGARCH models generalize the ARIMA and integrated GARCH (IGARCH) to include non-integer (fractional) differencing. In recent times, LM memory has been observed both in the mean and volatility in precious metals, the so-called dual LM, see e.g., Arouri et al. (2012) and Diaz (2016). Using ARFIMA–FIGARCH type models in the article by the first authors did not address structural breaks and heavy tailed error distributions while that by the second author only addressed the dual LM and asymmetry phenomena. Further, their LM analysis was not detailed.

In this study we attempt a more detailed and holistic approach, i.e., we address LM, structural breaks, asymmetry and heavy tailed distribution phenomenon in modelling platinum and palladium returns and volatility. We attempt to fill in the gaps by

- employing a wide spectrum of tests and methods which includes time domain, Fourier and wavelet domain techniques in exploring LM.
- distinguishing whether non-stationarity is spurious due to structural breaks or authentic.
- distinguishing whether non-stationarity is due to jumps in the mean or due to a trend.
- using a wider range of model selection and forecasting diagnostics.
- using a wider range of heavy tailed distributions.

In examining structural breaks we concentrate on validating whether the inherent LM is due to structural breaks, i.e., spurious or not. Most methods for testing the existence of structural breaks are based on out of sample forecasts and model comparison. On the other hand the two methods suggested by Shimotsu (2006) are advantageous in that they are unique in-sample tests for LM with good power and size. These tests are based on two notions, namely, the LM parameter estimate $\hat{d}$ from sub-samples of the full data set should be consistent with that of the full data set and that applying the $d\text {th}$ difference to an *I*(*d*) process should yield and *I*(0) process (based on KPSS test statistic). Although choosing a break fraction $\tau$ arbitrarily may be suboptimal, estimating it from the the data under the null hypothesis of no break existence would render $\hat{\tau }$ not to converge to a constant but to rather to a random variable which in turn adversely affect the asymptotic normality of the test statistic (Hassler and Olivares 2008). Different empirical multiple splitting scenarios are often arbitrarily carried out in practice before settling for one. Here in applying the former notion of the methods introduced by Shimotsu (2006) we split the full sample into sub-samples as in Arouri et al. (2012) who carried out a similar study.

Results from this method will assist in understanding if LM in the platinum and palladium returns are spurious or not. Lastly, we will compare different ARFIMA–FIGARCH type models under various distributional scenarios to find the models for platinum and palladium return and volatility series that best fit these data.

The outline of this paper is as follows. “Preliminary data exploration” section provides some preliminary data exploration aspects. “Long memory and structural breaks” section presents LM and structural breaks methods. “Volatility models” section discusses FIGARCH related volatility models. “Modelling of platinum and palladium returns series volatility” section gives empirical results of volatility models of the return series. “Conclusion” section gives the conclusion and further research work.

## Preliminary data exploration

The data used in this paper are daily closing platinum and palladium prices from February 1994 to June 2014, data is sourced from Matthey (2014). Both data series have 5237 data points. Log returns of price data used are defined as

1 $$\begin{aligned} r_t=ln\left( \frac{X_t}{X_{t-1}} \right) , \end{aligned}$$

where $X_t$ is the daily price at time *t* in days. As a point of departure we undertake a preliminary exploration of the return series of the two metals.

**Table 1:** Descriptive statistics of returns

Returns statistic	Platinum returns	Palladium returns
Q1	−0.0360	−0.0652
Q2	0.0049	0.0000
Q3	0.0450	0.0768
Mean	0.0000	0.0004
Kurtosis	11.9823	8.2040
Skewness	0.6577	0.7207

Descriptive statistics of the log returns of platinum and palladium are given in Table 1. Both returns are positively skewed indicating an asymmetric tail extending toward more positive values. Platinum returns have a higher kurtosis than the palladium ones while the skewness is vice-versa.

**Table 2:** Statistical tests of returns

Test	Platinum (P value)	Palladium (P value)
Kolmogorov–Smirnov	0.3655 (0.0001)	0.3569 (0.0001)
Jarque–Bera	31640.87 (0.0001)	15107 (0.0001)
Phillips–Perron (lags = 10)	−120.85 (0.01)	−220 (0.01)
Arch-LM (lags = 12)	1694.987 (<0.001)	1903.254 (<0.001)

**Fig. 1:**
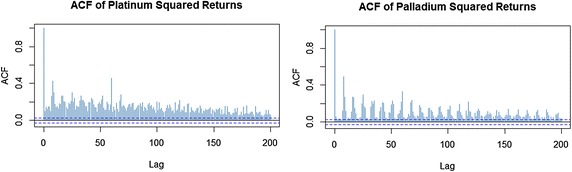
ACF of platinum and palladium squared returns

Jarque–Bera and Kolmogorov–Smirnov tests in Table 2 illustrates that the series are not Normally distributed. To test for unit roots, we use the Phillips–Perron test since it is robust to the presence of serial correlation and heteroskedasticity. Phillips–Perron test at truncation lag 10 shows that the returns are stationary in mean. The ARCH-test confirm that heteroskedasticity is inherent in both series. Further, the ACF plots of log squared returns in Fig. 1 show hyperbolic decay (unsummable ACFs), a phenomenon referred to as LM.

From these results, it is evident that these data are dominated by the stylized facts as well as LM. Since structural breaks usually induce spurious LM in financial time series, we discuss both LM and structural breaks in the next section.

## Long memory and structural breaks

A stationary time series process $X_t$ is a LM process if there exists a real number $0<H<1$ such that the ACF, denoted by $\rho (\tau )$, has a hyperbolic decay rate of the form $\lim _{x\rightarrow \infty }\rho (\tau )=C^{2H-2},$ where $C>0$ is a finite constant and *H* is the Hurst exponent (Hurst 1951). In LM literature, the parameter *d*, called the long range dependence (long memory) parameter is associated to the Hurst exponent with the relationship, $d=H-1/2$. Although the ARFIMA model is stationary and invertible for *d* in the range $-1/2<d<1/2$ evidence of precious metals exhibiting strong persistence ($0<d<1/2$) as opposed to intermediate persistence (antipersistence) ($-1/2<d<0$) is well documented in the literature, see e.g., Diaz (2016). The spectral density of a LM process will satisfy $f(\omega )=C|\omega |^{-2d}$, $0<d<1/2$. It is well known that this phenomenon can be spuriously induced by structural breaks. In this section we firstly dwell on LM and further elaborate on tests for structural breaks which confirm whether the inherent LM is authentic or spurious.

### Long memory estimation methods

In the literature, methods for estimating the long range dependence parameter are divided into three classes, namely heuristic, semi-parametric and maximum likelihood estimation (MLE) method. Heuristic (variance-type) methods are easy to compute and interpret but are both not accurate and robust. However, they are useful to test if LM exists and to obtain an initial estimate of *d* (or *H*). While on the other hand both semi-parametric and MLE methods give more accurate estimates, parametric methods require prior knowledge of the true model which infact is always unknown. For a comparative study of these classes of methods see Boutahar et al. (2007). In the following sub sections, we discuss these methods.

### Time domain estimation methods

In time domain analysis, a widely used heuristic method in estimating the Hurst exponent is the rescaled range estimator (*R*/*S*)(*n*) developed by Hurst (1951) and formerly introduced by Mandel (1971) in finance. This is mainly due to its simplicity and easy to estimate and interpret. For further details on this estimator see a paper by Kale and Butar (2010). The conclusions of Kristoufek and Lunackova (2013) and other authors in this field have recommended that this estimator must not be used in isolation, but rather be used in conjunction with other tests. Other time domain methods include aggregated variance, differenced aggregated variance and the aggregated absolute value estimators which are discussed by Teverovsky and Taqqu (1997) and Taqqu et al. (1995). The aggregated absolute value estimator only differ to aggregated variance one in that, instead of computing the sample variance the sum of absolute values of aggregated series is used. Another method very similar to this method that allows estimating the fractal dimension *D* such that $D=1-H$ for self-similar processes was suggested by Higuchi (1988). Also, another variance-type estimator based the variance of residuals was suggested by Peng et al. (1994). The differenced aggregated variance should be used together with the aggregated variance as the former can distinguish non-stationarity due to jumps in the mean from the one due to a slowly declining trend.

A desirable statistic that is often employed by analysts is the Kwiatkowsi, Phillips, Schmidt and Shin (KPSS) statistic (Kwiatkowski et al. 1992) because of its multifaceted diagnostic appeals, namely,

- The above authors suggested it for testing for unit roots in the economic time series, i.e., testing for both level-nonstationarity and trend nonstationarity.
- Lee and Schmidt (1996) used it to distinguish between short and LM processes.

Thus this statistic is applicable both in the short memory and LM frameworks.

The KPSS statistic is defined as

2 $$\begin{aligned} \eta = \frac{1}{T^2 \hat{\sigma _T}^2(q)} \sum _{\forall t}S_t^2, \quad t=1,\ldots ,T, \end{aligned}$$

where $S_t$ is the partial sum $\sum \nolimits _{i=1}^{t} \hat{e_i}$, with $\{\hat{e_i}\}$ denoting the residuals of the regression model and $\hat{\sigma _T^2}(q)$ is the Newey (1987) residuals weighted variance based on Bartlett lag window weights, $\lambda (s,q)=1-s/(q+1)$. Note that for testing level-nonstationarity, the residuals are based on the model with constant (intercept) term only, and the KPSS statistic is denoted by $\eta _\mu$ while for trend nonstationarity against a LM alternative of unit root, the residuals are based on the model with both intercept and trend, and the KPSS statistic is denoted by $\eta _t$. Another statistic that is algebraically similar to the KPSS statistic is the rescaled variance statistic, (*V*/*S*) (Giraitis et al. 2003), although its main purpose is restricted to the LM framework, i.e., estimating *H*.

### Fourier and wavelet based estimation methods

In this section we consider Fourier based and wavelet based methods for estimating the LM parameter. We first dwell on the fourier based methods. These methods are the so-called frequency domain techniques based on the log of the periodogram (log-periodogram). Various fourier based LM parameter estimators have proliferated since Geweke and Porter-Hudak (1983) (GPH) first suggested one such log-periodogram estimator.

Given a fractionally integrated process, its spectral density is given by

3 $$\begin{aligned} f(\omega )=\left[ 2 sin(\omega /2) \right] ^{-2d}f_u(\omega ), \end{aligned}$$

where $\omega$ is the Fourier frequency, $f_u(\omega )$ is the spectral density and $u_t$ is a stationary short memory disturbance with a zero mean. The log periodogram regression is based on applying logarithms to the above spectral density as follows

4 $$\begin{aligned} ln[f(\omega _j)]=ln[f_u(0)]-d ln\left[ 4 sin^2\left( \frac{\omega _j}{2} \right) \right] +ln\left[ \frac{f_u(\omega )}{f_u(u)} \right] . \end{aligned}$$

This then becomes

5 $$\begin{aligned} ln[I(\omega )] = a + d ln\left[ 4 sin^2\left( \frac{\omega _j}{2} \right) \right] + \eta , \end{aligned}$$

which we can re-parameterise as

6 $$\begin{aligned} y_j = a + dx_j + \eta _j \end{aligned}$$

where $y_j=ln[I(\omega _j)]$ and $x_j = ln\left[ 4 sin^2\left( \frac{\omega _j}{2} \right) \right]$. The long range dependence parameter is estimated as

7 $$\begin{aligned} \hat{d}=\frac{N\bar{x}\bar{y} -\sum _{j=1}^m y_i x_i}{\left( \sum _{j=1}^m x_i^2 -n \bar{x}^2 \right) }, \end{aligned}$$

where $m=g(T)$ and this estimator is asymptotically Normally distributed, i.e.,

8 $$\begin{aligned} \hat{d} \sim N\left( d, \frac{\pi ^2}{6\sum _{j=1}^m (X_j -\bar{X})}\right) , \end{aligned}$$

and for $T\rightarrow \infty$ we get

9 $$\begin{aligned} \sqrt{m}(\hat{d}-d)\sim \left( 0, \frac{\pi ^2}{6\sum _{j=1}^m (X_j -\bar{X})}\right) . \end{aligned}$$

The parameter *m* must be selected such that $m=T^\nu$, for $0<\nu <1$. The above formulation assumes ordinary least squares (OLS) and hence, an OLS estimate is derived with error terms being independent and identically Guassian distributed.

Since the periodogram is an unbiased but inconsistent estimator of the spectrum, a consistent estimator can be achieved by smoothing it (use of lag windows or averaging). One such consistent estimator is the modified (boxed) periodogram. Actually, Robinson (1994) proved that the averaged periodogram estimator was consistent under very mild conditions. It involves dividing the log of the periodogram into equally spaced boxes and then averaging the values inside each of the boxes leaving out very low frequencies. Further, to address the scattered nature of the periodogram, a robustified least squares (least-trimmed squares of regression) which minimises approximately *T* / 2 smallest squared residuals can be employed.

Another method that is used in conjuction with the log periodogram regression is the Whittle estimator (Kunsch 1987; Robinson 1995). The Whittle estimator is based on the periodogram and involves the evaluation of

10 $$\begin{aligned} Q(\varvec{\theta }) = \int _{-\pi }^{\pi }\frac{I(\omega )}{f(\omega ;\varvec{\theta })}d\omega , \end{aligned}$$

where $I(\omega )$ is the periodogram

11 $$\begin{aligned} I(\omega ) = \frac{1}{2\pi N}\left| \sum _{i=1}^N X_j e^{ij\omega } \right| ^2, \end{aligned}$$

and $f(\omega ; \varvec{\theta })$ is the spectral density at frequency $\omega$ and $\varvec{\theta }$ denotes the vector of unknown parameters, i.e., *d* and the autoregressive moving average (ARMA) parameters.

The Whittle estimator is the value of $\theta$ which minimises the function *Q* under a fractional integrated model, *ARFIMA*(0, *d*, 0), where $\theta$ is the fractional integration parameter *d* or the Hurst exponent *H* (Shimotsu and Phillips 2005). This means that the Whittle estimator of $\theta$ is

12 $$\begin{aligned} \hat{\theta _T}= arg \min _{\theta \epsilon \Theta } Q(\theta ), \end{aligned}$$

where $$Q(\theta )$$ is

13 $$\begin{aligned} Q(\theta ) \approx \frac{1}{T}\sum _{t=1}^T \left[ ln(f(\omega _t;\theta )) + \frac{I(\omega _t)}{f(\omega _t;\theta )} \right] ,\quad \omega _t = \frac{2\pi t}{T}. \end{aligned}$$

The local Whittle estimator of *d* or $\hat{\theta }$ is known to have the limiting distribution (Baillie and Kapetanios 2007)

14 $$\begin{aligned} m^{\frac{1}{2}}\left( \hat{d} - d_0 \right) \rightarrow N\left( 0, \frac{1}{4}\right) , \end{aligned}$$

where $d_0$ denotes the true value of *d* and *m* represents the choice of bandwidth such that $m\le T^{4/5}$.

One and a half decade after the advent of the GPH Fourier based estimator, Abry and Veitch (1998) ushered in the wavelet methodology in estimating the LM memory parameter. Wavelet based estimators have desirable properties, i.e., they capture the scale-dependent properties of data directly via the coefficients of a joint scale-time wavelet decomposition, require very little assumptions of the data generating process, are asymptotically unbiased and efficient and are robust to deterministic trends. Thus it is recommended that time domain and fourier based methods should be complemented by wavelet based ones.

Testing for LM and estimating the LM parameter may not be adequate in addressing the LM memory phenomenon as the presence of structural breaks can result in spurious LM. Therefore we attend to this aspect in the next section.

### Structural breaks diagnosis

When LM is due to structural changes in data, it is referred to as spurious LM. A simple method that can be used to detect spurious LM is due to Shimotsu (2006). In this method, the series of returns is split into *b* sub-samples and for each sub-sample, LM parameter is estimated. If LM is due to structural breaks, then the LM parameter estimates from the sub-samples should differ significantly from that of the full sample. The null hypothesis is

$$\begin{aligned} H_0:d=d^{(1)}=d^{(2)}=\cdots =d^{(b)} \end{aligned}$$

against the alternative of structural change hypothesis, where $\hat{d}^{(a)}$ is the value of *d* from the *a*th sub-sample. The sample that is split into *b* sub-samples has

$$\begin{aligned} \hat{\varvec{d}_{b}} = \left( \begin{array}{c} \hat{d}-d_0 \\ \hat{d}^{(1)}-d_0 \\ \vdots \\ \hat{d}^{(b)}-d_0 \end{array} \right) , \end{aligned}$$

and

$$\begin{aligned} \varvec{A} = \left( \begin{array}{cccc} 1& \quad -1& \quad \cdots & \quad 0 \\ \vdots &\quad \vdots &\quad \ddots &\quad \vdots \\ 1& \quad 0& \quad \cdots &\quad -1\end{array} \right) , \end{aligned}$$

where $d_0$ is the true parameter and $\hat{d}$ is the parameter estimate of the total sample. Let

$$\begin{aligned} \varvec{\Omega } = \left( \begin{array}{cc} 1 &\quad \varvec{J}_{b}'\\ \varvec{J}_{b}& \quad b\varvec{I}_{b}\end{array} \right) , \end{aligned}$$

where $\varvec{I}_b$ is a $b \times b$ identity matrix, and $\varvec{J}_b$ is a vector of ones. The Wald statistic and the adjusted Wald statistic under $H_0$ are

$$\begin{aligned} W=4m\varvec{A} \hat{\varvec{d}_b} \left( \varvec{A}\varvec{\Omega } \varvec{A}'\right) ^{-1}\left( A \hat{\varvec{d}_b} \right) ', \end{aligned}$$

and

$$\begin{aligned} W_c=4m\left( \frac{c_{m/b}}{(m/b)} \right) \varvec{A} \hat{\varvec{d}_b} \left( \varvec{A}\varvec{\Omega } \varvec{A}'\right) ^{-1}\left( \varvec{A} \hat{\varvec{d}_b}, \right) ', \end{aligned}$$

respectively, where the correction $c_m$ is given by

$$\begin{aligned} c_m=\sum _{j=1}^m v_j^2,\quad v_j=logj-\frac{1}{m}\sum _{j=1}^m logj\quad for\quad m<T. \end{aligned}$$

Under $H_0$, both *W* and $W_c$ have an asymptotic $\chi ^2$ distribution with $n-1$ degrees of freedom.

For non-stationary processes, this test utilises the fact that if an *I*(*d*) process is differenced *d* times, the resulting time series is an *I*(0) process. Shimotsu (2006) proposed a test that uses the Phillips–Perron and the KPSS test. The first step in this test is to demean the series into

$$\begin{aligned} X_t-\mu _0=(1-L)^{-d_0}u_t1_{\{t\ge 1\}}. \end{aligned}$$

The mean of the process $X_t$ is estimated by the sample average $\bar{X}$ when $d_0<1$. The $d\text {th}$ differenced series becomes $\hat{u}_t=(1-L)^{d}(X_t-\hat{\mu }(\hat{d}))$, where *L* is the backward operator such that $LX_t=X_{t-1}$. We apply *KPSS* test to $\hat{u}_t$. In the next Section, we discuss LM volatility models.

## Volatility models

Consider an ARFIMA model of the form

15 $$\begin{aligned} \phi (L) (1-L)^d X_j=\theta (L) \epsilon _j, \end{aligned}$$

where $\epsilon _t$ is a white noise process, $\phi (L)=1-\phi _1L-\phi _2L^2-\cdots -\phi _pL^p$ and $\theta (L)=1+\theta _1L+\theta _2L^2+\cdots +\theta _qL^q$. The assumption of constant variance is used mostly in time series analysis. In some cases, particularly financial time series, the volatility is not constant (heteroskedastic) and thus there are models proposed in literature to address this phenomenon. GARCH models are mostly used to explain volatility clustering and heteroskedasticity. The GARCH(*m*, *s*) model is defined as

16 $$\begin{aligned} a_t=\sigma _t \epsilon _t, \quad \sigma _t^2 = \alpha _0 + \sum _{i=1}^m \alpha _i a_{t-i}^2 + \sum _{j-1}^s \beta _j \sigma _{t-j}^2, \end{aligned}$$

where $\{\epsilon _t\}$ is a sequence of *i*.*i*.*d* random variables, i.e., $E(\epsilon _t)=0$ and $Var(\epsilon _t)=1$ with $\alpha _0 >0, \alpha _i\ge 0, \beta _j\ge 0$, $\sum _{i=1}^{max(m,s)} (\alpha _i + \beta _i) <1$ and $a_t$ is the mean corrected returns $a_t=r_t - \mu _t$, and $\mu _t$ is the mean of the return series. GARCH models are better understood if they are in an ARMA form as follows

17 $$\begin{aligned} a_t^2 = \alpha _0 + \sum _{i=1}^{max(m,s)} (\alpha _i + \beta _i)a_{t-i}^2 + \eta _t - \sum _{j=1}^s \beta _j \eta _{t-j}, \end{aligned}$$

where $\eta _t = a_t^2 - \sigma _t^2$ and $\{\eta _t\}$ is a martingale difference. Expression 17 satisfies the $ARCH(\infty )$ representation

$$\begin{aligned} \sigma _t^2 = \frac{\omega }{\beta (1)}+\Psi ^{GA}(L)a_t^2 =\frac{\omega }{\beta (1)}+\sum _{i=1}^{\infty }\psi _i^{GA}a_{t-i}^2, \end{aligned}$$

where $\Psi ^{GA}(L)=[\beta (L)-\phi (L)]/\beta (L)=\alpha (L)/\beta (L)$ and coefficients $\psi _i^{GA}$ are defined recursively as $\psi _1^{GA}=\phi _1-\beta _1$ and $\psi _i^{GA}=\beta _1\psi _{i-1}^{GA}$, for $i\ge 2$.

If the AR polynomial in the above has unit roots such that $\sum _{i=1}^{max(m,s)} (\alpha _i + \beta _i) \approx 1$, the resulting model becomes an integrated GARCH (IGARCH) model. A key feature of this model outlined in Tsay (2002) is that the impact of past squared shocks $\eta _{t-i} = a_{t-i}^2 - \sigma _{t-i}^2$ on $a_t^2$ are persistent. When the return series contains LM, its ACF is not summable as it declines hyperbolically as the lag increases. In this case, the fractional IGARCH (FIGARCH) model is used.

The FIGARCH model is characterised by a volatility persistence shorter than an IGARCH model but longer that the GARCH model. The FIGARCH model is obtained by extending the IGARCH model and allowing the integration factor to be fractional. The FIGARCH(*p*, *d*, *q*) is defined

18 $$\begin{aligned} r_t = \mu _t + a_t, \quad \sigma _t^2 = \omega (1-\beta (L))^{-1}+\left\{ 1-(1-\beta (L))^{-1} \phi (L)(1-L)^d \right\} a_t^2, \end{aligned}$$

where $\beta (L)=\beta _1L^1+\beta _2L^2+\cdots +\beta _pL^p$. The exponential FIGARCH (FIEGARCH) model is defined as

19 $$\begin{aligned} ln(\sigma _t^2)=\alpha _0+\frac{1-\sum _{i=1}^p \alpha _i L^i}{1-\sum _{j=1}^q \beta _j L^j}(1-L)^{-d} g(\epsilon _{t-1}), \end{aligned}$$

where

20 $$\begin{aligned} g(\epsilon _{t-1})=\theta \epsilon _{t-1}+\gamma [|\epsilon _{t-1}|-E(|\epsilon _{t-1}|)] \quad \forall t \in \mathbb {Z}, \end{aligned}$$

and $\gamma$ is the rate at which innovations deviate from the mean. FIEGARCH processes models more than LM and volatility, they also explain volatility clusters and asymmetry. Thus these models offer better modeling capability than FIGARCH ones as they don’t suffer from FIGARCH drawbacks since the variance under FIEGARCH is defined in terms of the logarithm function.

The fractional integrated asymmetric power ARCH (FIAPARCH) process increases the flexibility of the conditional variance specification by allowing

1. An asymmetric response of volatility to positive and negative shocks,
2. The data to determine the power of returns for which the predictable structure in the volatility pattern is strongest, and
3. Long range volatility dependence.

A simple FIAPARCH (1, *d*, 1) model is given by

21 $$\begin{aligned} (1-\phi L)(1-L)^d f(a_t)=\alpha _0 +[1-\beta (L)]a_t, \end{aligned}$$

where

22 $$\begin{aligned} f(\epsilon _t)=[|a_t|-\gamma a_t]^{\delta }, \end{aligned}$$

$\gamma$ is the leverage parameter defined in $-1<\gamma <1$, $\delta$ is the parameter for the power term, $|\phi |<1$, $\alpha _0>0$ and $0\le d\le 1$. This process would reduce to the FIGARCH process for $\gamma =0$ and $\delta =2$.

The hyperbolic GARCH (HYGARCH) model introduced by Davidson (2004) has the GARCH model and FIGARCH model as special cases. It is covariance stationary, similar to the GARCH model and has hyperbolic decay impulse response coefficients similar to the FIGARCH model. The HYGARCH process is obtained by

23 $$\begin{aligned} \phi (L)\left( (1-\tau )+\tau (1-L)^d \right) a_t^2=\alpha _0+\beta (L)\eta _t. \end{aligned}$$

When $\tau =0$ and $d=0$, the model is GARCH and when $\tau =1$, the model is FIGARCH. To further understand this model, we can re-write is as

24 $$\begin{aligned} \sigma _t^2&= \frac{\alpha _0}{\beta (1)}+\Psi ^{HY}(L)a_t^2,\nonumber \\&= \frac{\alpha _0}{\beta (1)}+\sum _{i=1}^\infty \psi _i^{HY}a_{t-i}^2, \end{aligned}$$

where

25 $$\begin{aligned} \Phi ^{HY}(L)=\tau \Psi ^{FI}(L)+(1-\tau )\Psi ^{GA}(L), \end{aligned}$$

with $\Psi ^{FI}(L)=1-[(1-L)^d\phi (L)/\beta (L)]$ and coefficients $\psi _i^{FI}$ are given as $\psi _1^{FI}=d+\phi _1-\beta _1$ and $\psi _i^{FI}=\beta _1\psi _{i-1}^{FI}+(f_i -\phi _1)(-g_{i-1})$, for $i\ge 2$ and both $f_i$ and $g_i$ are functions of differencing parameter *d* and thus it follows that

26 $$\begin{aligned} \psi _i^{HY}=\tau \psi _i^{FI}+(1-\tau )\psi _i^{GA}. \end{aligned}$$

In the following Section, we discuss the application results from modeling the platinum and palladium return series using these models.

## Modelling of platinum and palladium returns series volatility

In this section we discuss the results from structural breaks diagnosis. This will assist with the identification of breaks inherent in data. We then discuss the results from LM tests to examine LM properties of the series. Lastly, we report of the results of volatility models used under various distributional scenarios and the evaluation of forecasts.

### Structural breaks diagnosis

In structural breaks diagnosis, we used a method introduced by Shimotsu (2006) which tests parameter consistency using sub-samples methodology. The results of this test are shown in Tables 3 and 4 for platinum and palladium returns, respectively. For this method, we split the sample into sub-samples and for each of the sub-samples selected, we obtain estimates of *d*. The long range dependence parameter estimates for the sub-samples, $\hat{d}_2$ and $\hat{d}_4$ which are the averages of splitting the sub-sample into 2 and 4 samples respectively. We used the Wald test statistic on $\hat{d}_2$ and $\hat{d}_4$ to test parameter consistency in long range dependence parameters. Chi-square critical values $\chi _{0.95}^2(1)$ = 3.84 and $\chi _{0.95}^2(4)$ = 7.82 were used as cut off values for testing the significance of $d_2$ and $d_4$ at the 5% level of significant, respectively.

**Table 3:** Test results of platinum squared returns

m	$\hat{d}$	$\hat{d_2}$	$\hat{d_4}$	$W_2$	$W_4$	KPSS	P value (KPSS)
500	0.0125	0.0095	−0.0494	0.6151	5.6580	0.0077	0.1000
1000	0.1275	0.0125	0.0095	7.9680	19.4500	0.0184	0.1000
1500	0.0763	−0.0016	0.0091	0.0001	10.9500	0.0185	0.1000
2000	0.0781	0.1275	0.0125	22.6700	31.5300	0.0089	0.1000
2500	0.0670	0.1156	0.0120	38.5800	15.0100	0.0077	0.1000
3000	0.0722	0.0763	−0.0016	3.0550	11.5700	0.0131	0.1000
3500	0.0672	0.1054	0.1547	16.3000	46.6100	0.0250	0.1000
4000	0.1085	0.0781	0.1275	2.8320	30.0600	0.0093	0.1000
4500	0.1076	0.0712	0.1175	5.3880	33.0400	0.0153	0.1000
5000	0.1076	0.0670	0.1156	8.0600	53.4300	0.0145	0.1000

**Table 4:** Test results of palladium squared returns

m	$\hat{d}$	$\hat{d_2}$	$\hat{d_4}$	$W_2$	$W_4$	KPSS	P value (KPSS)
500	−0.1426	−0.1589	−0.1550	1.4070	0.1183	0.0942	0.1000
1000	0.0839	−0.1426	−0.1589	43.1200	3.7230	4.5200	0.1000
1500	0.0947	−0.0862	−0.1659	24.3500	67.1800	2.4780	0.1000
2000	0.0769	0.0839	−0.1426	1.7850	81.4900	6.4480	0.1000
2500	0.0755	0.0943	−0.1140	4.0330	50.0400	3.5400	0.1000
3000	0.0770	0.0947	−0.0862	4.2810	31.0300	2.1110	0.1000
3500	0.0806	0.1183	0.0938	8.3180	9.2730	1.6800	0.1000
4000	0.0755	0.0769	0.0839	0.0544	1.9680	1.6640	0.1000
4500	0.0721	0.0764	0.0918	0.5930	5.7750	1.3290	0.1000
5000	0.0762	0.0755	0.0943	0.0134	6.6750	0.9774	0.1000

From Table 3, it is evident that the platinum return series contain breaks as the long range dependence parameter is not consistent between sub-samples and hence, between samples and the full data set. This is further shown by the rejection of parameter consistency by $W_2$ and $W_4$ tests. The KPSS test statistic does not reject the presence of LM.

Results of palladium return series in Table 4 show that the series contain breaks as well. However, the Wald test statistics $W_2$ and $W_4$ do not reject parameter consistency in as many sub-samples as seen in platinum return series results in Table 3. Further, the KPSS statistic does not reject the presence of LM as well. This is indicative of the fact that not all LM maybe spurious, i.e., due to structural breaks. In the next sub section, we further carry out more tests for LM and estimate the long range dependence parameter using different estimation methods.

### Long memory tests

In LM testing, we fitted different LM tests to the squared log returns of platinum and palladium prices. The Hurst exponent results of LM tests are shown in Table 5 for both platinum and palladium squared log returns.

**Table 5:** Platinum and palladium log squared returns LM tests

Method	Hurst	Standard error	t value	P value
Platinum log squared returns				
Aggregated variance method	0.9358 (0.4358)	0.0421	22.2319	<0.0001
Differenced aggregated variances	1.1907 (0.6907)	0.1813	6.5684	<0.0001
Aggregated absolute value method	0.9909 (0.4909)	0.0253	39.2360	<0.0001
Higuchi method	0.9739 (0.4739)	0.0358	27.1544	<0.0001
Peng method	0.6836 (0.1836)	0.1127	6.0681	<0.0001
R/S method	0.6667 (0.1667)	0.0754	8.8486	<0.0001
Periodogram method (GPH)	0.9665 (0.4665)	0.0382	25.2618	<0.0001
Boxed (modified) periodogram method	0.8313 (0.3313)	0.0463	17.9453	<0.0001
Wavelet estimator	0.5248 (0.0248)	0.1005	5.2199	0.0034
Whittle estimator	0.6080 (0.1080)	0.0089	68.2000	<0.0001
Palladium log squared returns				
Aggregated variance method	0.9340 (0.4340)	0.0606	15.4258	<0.0001
Differenced aggregated variances	0.9175 (0.4175)	0.1656	5.5416	<0.0001
Aggregated absolute value method	0.9625 (0.4625)	0.0342	28.1158	<0.0001
Higuchi method	0.9754 (0.4754)	0.0380	25.6083	<0.0001
Peng method	0.5451 (0.0451)	0.1256	4.3386	<0.0001
R/S method	0.4038 (−0.0962)	0.1471	2.7450	0.0087
Periodogram method (GPH)	0.9103 (0.4103)	0.0384	23.7393	<0.0001
Boxed (modified) periodogram method	0.7707 (0.2707)	0.0448	17.2098	<0.0001
Wavelet estimator	0.6145 (0.1145)	0.1510	4.0691	0.0096
Whittle estimator	0.5779 (0.0779)	0.0088	65.4000	<0.0001

On platinum squared log returns, all of the tests used suggest LM as all the P values are less than 0.01. Note that the differenced aggregated variances method violates the condition $0<H<1$. This should not be a concern as its main purpose is to distinguish nonstationarity due to jumps ($H\approxeq 0.5$) to that due to actual trend ($H\gg 0.5$). So in this case, trend is not due to jumps in the data. It is clear that platinum squared returns have high persistence and it appears they could be explained by a fractionally integrated model.

Like platinum, palladium log squared returns also suggest a high degree of LM as confirmed by very low P values, hence they can be explained by a fractionally integrated model. In the next sub section, we fit LM mean models and conditional volatility models on both platinum and palladium return series to investigate the dual LM of mean returns and volatility.

### Empirical results of volatility models

To explain the dual LM of the mean and volatility of platinum and palladium return series, we fitted ARFIMA–FIGARCH type models under heavy tailed error distributions including the Normal distribution. We used an ARFIMA model for modelling squared log returns and for volatility we used FIGARCH, FIEGARCH, FIAPARCH and HYGARCH models under heavy tailed error distributions bench marking them with the Normal distribution. Distributions considered are the Normal, Student, Generalized extreme distribution (GED), and the skewed Student distribution.

Parameter estimation results are shown in Tables 6, 7, 8 and 9. Let $d_m$ denote LM paramater in the mean model. For all the models, the long range dependence parameter of the ARFIMA model is negative ($-1/2<d_m<0$) indicating anti-persistence (intermediate persistence). This illustrates that log returns of both platinum and palladium are mean reverting and hence, will revert to the mean overtime. Let $d_v$ denote LM paramater in the volatility model. For volatility the long range dependence parameter is positive ($0<d_v<1$) and shows strong LM. This confirms the results by other authors (Arouri et al. 2012), platinum shows high persistence.

**Table 6:** ARFIMA–FIGARCH parameter estimation of models

Parameters	Normal platinum	Student	GED	Skewed student	Normal palladium	Student	GED	Skewed student
Cst(M)	0.0002**	0.0001***	0.0001**	0.0003***	0.0001	0.0001		0.0002***
$d_m$	−0.1027***	−0.0893**	−0.0838	−0.5276***	−0.1019***	−0.0601*	−0.4513***	−0.5062***
AR(1)	0.4062***	0.4158***	0.4193***	0.1356***	0.3908***	0.4217***	0.1172***	0.0887
MA(1)	−0.9327***	−0.9353***	−0.9361***	0.0699*	−0.9225***	−0.9302***	0.0191	0.0818*
Cst(V)	0.0013**	0.0011*	0.0011*	0.0006	0.0095	0.0104*	0.0087***	0.0085*
$d_v$	0.6492***	0.6486***	0.6473***	0.6545***	0.7227***	0.7040***	0.6419***	0.6258***
ARCH($\alpha _1$)	0.3686***	0.3567***	0.3543***	0.2138***	0.3907***	0.3678***	0.3088***	0.3094***
GARCH($\beta _1$)	0.8721***	0.8730***	0.8720***	0.8680***	0.9016***	0.8986***	0.8712***	0.8708***
Akaike	–2.4717	–2.4720	–2.4715	–2.4017	−1.2415	−1.2534	−1.1814	−1.1740
Schwarz	–2.4591	–2.4578	–2.4574	–2.3860	−1.2289	−1.2393	−1.1688	−1.1583
ARCH-LM	4.0565**	4.7917***	4.6751***	4.5570**	0.97785	4.1599**	7.8780***	31.069***

*, ** and *** represent the significant level at 10, 5 and 1% levels respectively

**Table 7:** ARFIMA–FIEGARCH parameter estimation of models

Parameters	Normal platinum	Student	GED	Skewed student	Normal palladium	Student	GED	Skewed student
$d_m$	−0.4668***	−0.4012***	−0.3903***	−0.0488	−0.1190***	−0.4738***	0.0010	0.4776***
AR(1)	−0.2576***	−0.0012	0.0300	0.4407***	0.3981***	0.0399	0.4509***	0.0857*
MA(1)	0.3411***	0.1230	0.0863	−0.9404***	−0.9183***	0.1032***	0.9381***	0.0711*
$d_v$	0.8975***	0.9173***	0.9173***	0.6623***	0.2624***	0.9061***	0.8884***	0.9088***
ARCH($\alpha _1$)	1.1344***	1.2337***	1.2557***	−0.6664***	−0.8026***	−0.3899***	0.5881***	0.4803***
GARCH($\beta _1$)	−0.8946***	−0.8977***	−0.9047***	0.8725***	0.9845	−0.2092*	0.0432	0.2842***
EGARCH($\theta _1$)	0.0234***	0.0645***	0.0433***	0.0414	0.0212	−0.0092	0.0387*	0.0061
EGARCH($\theta _2$)	0.2187***	0.2279***	0.2122***	0.1367	0.4046***	0.4426***	0.4089***	0.3307***
Akaike	–2.2483	–2.2809	–2.2885	–2.4602	−1.1975	−1.1087	−1.2389	−1.1452
Schwarz	–2.2358	–2.2668	–2.2743	–2.4413	−1.1817	−1.0945	−1.2216	−1.1278
ARCH-LM	0.8213	14.202***	14.175***	1.6987	4.0757**	3.5115**	0.60530	2.2058

*, ** and *** represent the significant level at 10, 5 and 1% levels respectively

**Table 8:** ARFIMA–FIAPARCH parameter estimation of models

Parameters	Normal platinum	Student	GED	Skewed student	Normal palladium	Student	GED	Skewed student
Cst(M)	0.0001	0.0001	0.0001	0.0003***	0.0001	0.0001	0.0001	0.0001
$d_m$	−0.0793***	−0.0556	−0.0410	−0.5176***	−0.1001***	−0.0582***	−0.00968	−0.0587**
AR(1)	0.3920***	0.4053***	0.4137***	0.1308***	0.3884***	0.4202***	0.4516***	0.4186***
MA(1)	−0.9327***	−0.9361***	−0.9381***	0.0687*	−0.9229***	−0.9306***	−0.9382***	−0.9306***
Cst(V)	0.0041	0.0043*	0.0046*	0.0005	0.0106	0.0156*	0.0166*	0.0150*
$d_v$	0.6512***	0.6462***	0.6378***	0.6432***	0.7306***	0.6980***	0.6877***	0.6987***
ARCH($\alpha _1$)	0.3974***	0.3929***	0.3919***	0.2325***	0.3872***	0.3703***	0.3551***	0.3716***
GARCH($\beta _1$)	0.8743***	0.8744***	0.8718***	0.8607***	0.9035***	0.8973***	0.8900***	0.8974***
APARCH($\gamma _1$)	−0.2928***	−0.3716***	−0.4169*	−0.2012*	−0.0863*	−0.0859	−0.09485	−0.0914
APARCH($\delta$)	1.8067***	1.7569***	1.7309***	2.0904***	1.9933***	1.9222***	1.9228***	1.9234***
Akaike	–2.4773	–2.4783	–2.4781	–2.4030	−1.2420	−1.2532	−1.2589	−1.2533
Schwarz	–2.4616	–2.4610	–2.4608	–2.3841	−1.2262	−1.2359	−1.2416	−1.2344
ARCH-LM	2.2572	2.6098*	2.7374*	2.8892*	0.67543	3.8257*	2.6699*	3.5548**

*, ** and *** represent the significant level at 10, 5 and 1% levels respectively

**Table 9:** ARFIMA–HYGARCH parameter estimation of models

Parameters	Normal platinum	Student	GED	Skewed student	Normal palladium	Student	GED	Skewed student
$d_m$	−0.0802***	−0.4125***	−0.3947***	−0.4247***	−0.4988***	−0.4660***	−0.4508***	−0.4691***
AR(1)	0.3788***	0.0254	0.0684	0.0899**	−0.3287***	0.0543	0.1039***	0.0521
MA(1)	−0.9355***	0.0985*	0.0540	0.0587	0.4119***	0.0889*	0.0294***	0.0935***
$d_v$	0.8199***	0.7930***	0.7968***	0.7983***	0.7266***	0.6475***	0.6741***	0.6474***
ARCH($\alpha _1$)	0.2981***	0.1091***	0.1086***	0.0960***	0.3045***	0.2995***	0.2968***	0.3002***
GARCH($\beta _1$)	0.9142***	0.909***2	0.9088***	0.9113***	0.8925***	0.8787***	0.8799***	0.8790***
Akaike	–2.4590	–2.3467	–2.3547	–2.3710	−1.1248	−1.1650	−1.1786	−1.1649
Schwarz	–2.4480	–2.3341	–2.3421	–2.3568	−1.1138	−1.1524	−1.1660	−1.1508
ARCH-LM	1.9986	1.4914	0.72470	0.72483	0.064012	28.207***	5.9269***	27.107***

*, ** and *** represent the significant level at 10, 5 and 1% levels respectively

#### Model selection results for platinum

Based on the Akaike information criterion, the best model is the ARFIMA–FIAPARCH under the Student distribution. However, the ARCH-effect is slightly significant (*). Based on the Schwarz information criterion, the best model is the ARFIMA–FIAPARCH under the Normal distribution and has no ARCH-effect. Although the ARFIMA–FIEGARCH under the Skewed Student distribution and ARFIMA–HYGARCH under the Normal distribution were not selected based on the two information criteria they have no ARCH-effect.

#### Model selection results for palladium

In the case of palladium based on both Akaike and Schwarz information criteria selected the ARFIMA–FIAPARCH under the GED. However, the ARCH-effect is slightly significant (*). Although the ARFIMA–FIEGARCH under the GED and ARFIMA–HYGARCH under the Normal distribution were not selected based on the two information criteria they have no ARCH-effect.

The results of the two metals agree with the results of Diaz (2016) who found that platinum and palladium returns volatility are characterized by asymmetric response to negative and positive shocks as explained by the FIAPARCH model. From the results for models, $\gamma<$0 which illustrates that positive shocks have relatively more impact on volatility than negative shocks. Thus, although these metals respond to negative and positive news the same, positive news have a higher impact and thus making these metals a good investment vehicle as outlined in Arouri et al. (2012). We discuss forecasting performance of these models in the following sub section.

### Forecast evaluation methods

Evaluation of forecasts for models is important as it helps us understand the forecasting accuracy of the models estimated. There are a number of forecasts evaluation measures available in the literature. For our analysis, we used three measures commonly used in literature, namely the mean square error (MSE), the mean absolute error (MAE) and the Theil Inequality Coefficient (TIC). These measures are defined

$$\begin{aligned} MSE & = \frac{1}{n}\sum _{t=1}^n(\sigma _t -\hat{\sigma }_t)^2,\\ MAE & = \frac{1}{n}\sum _{t=1}^n|\sigma _t -\hat{\sigma }_t| \end{aligned}$$

and

$$\begin{aligned} TIC=\frac{\sqrt{1/n\sum _{t=1}^n(\sigma _t -\hat{\sigma }_t)^2}}{\sqrt{1/n \sum _{t=1}^n \sigma _t^2}+\sqrt{1/n \sum _{t=1}^n \hat{\sigma _t}^2}}, \end{aligned}$$

where *n* is the number of forecasts, $\sigma _t$ is the observed volatility and $\hat{\sigma }_t$ is the predicted conditional volatility at time *t*. The best model must exhibit least prediction error as given by the three measures.

Another popular method used for assessing forecasting performance of volatility models is the Mincer–Zarnowitz regression defined as

$$\begin{aligned} \tilde{\sigma }_t^2=\alpha +\beta \hat{\sigma }_t^2+u_t, \quad t=12,...,T, \end{aligned}$$

where $\tilde{\sigma }_t^2$ is the observed volatility as measured by squared innovations and $\hat{\sigma }_t^2$ is the predicted volatility. If the conditional volatility model is correctly specified and $\tilde{\sigma }_t^2$ is unbiased for the true variance then the parameters will take values $\alpha =0$ and $\beta =1$. This then suggest that the observed volatility will completely be explained by the predicted volatility. An $R^2$ value from this regression model compares predictive ability of volatility models. The Mincer–Zarnowitz regression results are shown in Tables 10, 11, 12 and 13. In this this regression the significance of both (alpha) and slope (beta) are tested. For all the models used, the null hypothesis for zero intercept is rejected at 5% level of significance. This tells us that the models will underestimate or overestimate the volatility to some extent and thus we would need to adjust the forecasts with calculated intercept values.

Tables 10, 11, 12 and 13 show forecast evaluation results. For platinum return series, the MSE gives low prediction errors for all models except ARFIMA–FIEGARCH which has slightly high errors. Further, based on the MAE, the ARFIMA–FIAPARCH under the Normal and Student distribution and the ARFIMA–HYGARCH model under the Normal distribution gives less prediction errors. Lastly, based on the TIC, the ARFIMA–FIEGARCH under Student distribution gives less prediction error

For palladium, based on the MSE the ARFIMA–FIAPARCH under the Normal distribution performs best. Further, the ARFIMA–FIAPARCH under Student, Skewed Student and GED distributions give less errors. Lastly, based on the TIC, the ARFIMA–FIEGARCH under the Normal distribution gives less prediction error. Hence this confirms the selection of ARFIMA–FIAPARCH models under Student and GED error distributions as good models since it it evident from the MAE evaluation measure.

**Table 10:** ARFIMA–FIGARCH forecast evaluation

Parameters	Normal platinum	Student	GED	Skewed student	Normal palladium	Student	GED	Skewed student
MSE	0.0003	0.0003	0.0003	0.0003	0.0037	0.0037	0.0038	0.0038
MAE	0.0082	0.0082	0.0083	0.0087	0.0256	0.0257	0.0272	0.0269
TIC	0.6515	0.6517	0.6517	0.6331	0.6308	0.6407	0.6356	0.6391
Alpha (MZ)	0.0002	0.0002	0.0002	0.0006	−0.0029	−0.0026	−0.0022	−0.0029
Beta (MZ)	1.2062	1.1974	1.1948	1.0028	1.3566	1.3449	1.1784	1.2280
$R^2$ (MZ)	0.0689	0.0670	0.0665	0.0551	0.1443	0.1274	0.0972	0.1000

**Table 11:** ARFIMA–FIEGARCH forecast evaluation

Parameters	Normal platinum	Student	GED	Skewed student	Normal palladium	Student	GED	Skewed student
MSE	0.0004	0.0004	0.0004	0.0004	0.0037	0.0043	0.0037	0.0039
MAE	0.0097	0.0115	0.0107	0.0085	0.0260	0.0362	0.0284	0.0327
TIC	0.6012	0.5626	0.5773	0.6657	0.5460	0.5479	0.5959	0.5725
Alpha (MZ)	0.0034	0.0035	0.0037	0.0028	−0.0005	0.0055	0.0026	0.0019
Beta (MZ)	0.5231	0.3826	0.4132	0.7664	1.2041	0.5204	0.8858	0.7139
$R^2$ (MZ)	0.0317	0.0320	0.0307	0.0220	0.1277	0.0832	0.1067	0.0861

**Table 12:** ARFIMA–FIAPARCH forecast evaluation

Parameters	Normal platinum	Student	GED	Skewed student	Normal palladium	Student	GED	Skewed student
MSE	0.0003	0.0003	0.0003	0.0003	0.0036	0.0037	0.0038	0.0037
MAE	0.0081	0.0081	0.0082	0.0087	0.0257	0.0255	0.0255	0.0255
TIC	0.6699	0.6721	0.6727	0.6384	0.6186	0.6383	0.6475	0.6369
Alpha (MZ)	0.0004	0.0005	0.0005	−0.0009	−0.0034	−0.0036	−0.0032	−0.0036
Beta (MZ)	1.2716	1.2550	1.2415	1.1667	1.3477	1.4073	1.4054	1.4052
$R^2$ (MZ)	0.0634	0.0584	0.0554	0.0510	0.1587	0.1401	0.1270	0.1416

**Table 13:** ARFIMA–HYGARCH forecast evaluation

Parameters	Normal platinum	Student	GED	Skewed student	Normal palladium	Student	GED	Skewed student
MSE	0.0003	0.0003	0.0003	0.0003	0.0038	0.0038	0.0038	0.0038
MAE	0.0081	0.0088	0.0087	0.0092	0.0268	0.0270	0.0268	0.0271
TIC	0.6581	0.6319	0.6351	0.6152	0.6345	0.6363	0.6403	0.6353
Alpha (MZ)	0.0007	0.0013	0.0013	0.0014	−0.0023	−0.0025	−0.0014	−0.0025
Beta (MZ)	1.2053	0.9064	0.9233	0.8067	1.2148	1.1995	1.1788	1.1942
$R^2$ (MZ)	0.0737	0.0491	0.0495	0.0470	0.1076	0.0998	0.0966	0.0998

For the selected models the platinum model has intercept estimate of 0.0005 and the palladium model has intercept estimate of −0.00032, hence the platinum model underestimates volatility while the palladium model overestimates volatility. The null hypothesis of a unit slope is not rejected at 5% level of significance for all models. This tells us that our forecasts from the models explains the observed values. In summary, the ARFIMA–FIGARCH type models under heavy tailed error distributions show an improvement of forecasts as compared to the assumption of Normally distributed errors, and further ARFIMA–FIAPARCH models proved to explain platinum and palladium return series better under non Normal error distributions.

## Conclusion

With the current South African economic conditions and volatile commodity markets, it is of interest to understand the distribution of platinum group metals and inherent volatility overtime. As it is widely known in literature that financial returns do not follow Normal distributions, we used different heavy tailed error distributions.

Recently LM has been a phenomena of interest in econometrics and financial markets. LM is summarized by the long range dependence parameter. Since spurious LM can also result from structural breaks in data, we used the sub-sample methodology to test long range dependence parameter consistency to establish whether the LM is spurious or not. From the results, we found that both platinum and palladium log squared returns contain structural breaks. This was identified by long range dependence parameter estimates not being consistent in sub-sample estimation. To further analyze LM, we used the fact that the $d\text {th}$ difference of an *I*(*d*) process should yield an *I*(0) process (based on KPSS test statistic.) This further confirmed results of high persistence in platinum and palladium as documented in the literature.

To understand and model volatility inherent in log squared returns of platinum and palladium, we fitted ARFIMA–FIGARCH related models under heavy tailed error distributions bench marking these distributions with the Normal distribution. These models are able to capture LM and the stylized facts in returns and volatility. In forecasting volatility using these models, adjustments from the Mincer–Zarnowitz regression needs to the factored in as these models will slightly underestimate/overestimate volatility.

Results from the paper points to the need for more empirical analysis on the platinum group metals. For further research, we will compare time varying ARFIMA–FIGARCH type models that will factor in structural breaks to structural breaks adjusted ARFIMA–FIGARCH type models.
